# Special Issue: Trends in Clinical Deep Brain Stimulation

**DOI:** 10.3390/jcm10020178

**Published:** 2021-01-06

**Authors:** Marcus L. F. Janssen, Yasin Temel

**Affiliations:** 1 Department of Clinical Neurophysiology, Maastricht University Medical Center, P. Debyelaan 25, 6229 HX Maastricht, The Netherlands; 2 School for Mental Health and Neuroscience, Faculty of Health, Medicine and Life Sciences, Maastricht University, Universiteitssingel 40, 6229 ER Maastricht, The Netherlands; y.temel@maastrichtuniversity.nl; 3 Department of Neurosurgery, Maastricht University Medical Center, P. Debyelaan 25, 6229 HX Maastricht, The Netherlands

Deep brain stimulation (DBS) has been successfully applied in several neurological and psychiatric disorders. A substantial number of patients suffering from a brain disorder either do not, or insufficiently, respond to pharmacological treatment. This results in increasing costs for public health systems and a growing burden for society. Fortunately, the number of approved indications for DBS keeps expanding, thereby improving the quality of life of many individuals. Nevertheless, defining the optimal target and stimulation paradigm for the individual patient remains a challenge. In this Special Issue, a series of twelve papers is presented by international leaders in the field on the current trends in clinical deep brain stimulation for a range of neurological and psychiatric disorders. 

One of the most common psychiatric disorders considered to be treated using DBS is depression. Unfortunately, recent randomized controlled trials show disappointing results of DBS for treatment-resistant depression (TRD). Contrary to these findings, the meta-analysis conducted by Hitti et al. shows that DBS is an effective treatment for TRD [1]. This promising finding should serve as an encouragement for future studies to optimize patient selection, stimulation settings and target selection. In line with this, Roet et al. also plead for a more personalized treatment approach for patients suffering from TRD [2]. They conclude that depression should not be considered as one disorder and patients should be subtyped. Target selection would depend on specific patient characteristics assessed by a variety of biomarkers, such as clinical characteristics and findings from (functional) imaging studies. The authors argue that postoperative monitoring using momentary assessment techniques could be helpful in optimizing DBS therapy for the individual patient suffering from major depressive disorder. Khairuddin et al. conducted an in-depth literature review on DBS treatment of the subcallosal cingulate in patients with TRD [3]. This review displays the immense differences in response and remission rates between studies. These differences might be overcome by a more personalized approach. The authors underline the complexity of evaluating treatment effects in this patient group. Important inroads are also being made in the understanding of DBS working mechanisms in the treatment of TRD using preclinical studies. Animal studies show distant stimulation effects in the limbic network and neuroplasticity, as well as modifications at the molecular level. 

In contrast to major depressive disorder, DBS for obsessive compulsive disorder (OCD) has the approval of the FDA as a humanitarian device exemption. The individual outcome of DBS, however, varies between patients. Generally, several pharmaceutical, as well as non-pharmaceutical, behavioral therapies are offered to patients before DBS surgery. The potential amplifying effect of these therapies in combination with DBS has insufficient attention. The review by Görmezoǧlu et al. highlights the need to better investigate the synergetic effects of cognitive behavioral therapy (CBT) and DBS in patients suffering from OCD [4].

Chronic pain is a debilitating neurological symptom which is difficult to treat. Its inherently chronic nature has a huge impact on the quality of life of the individual affected, as well as societal costs. From a pathophysiological perspective, it is not surprising that many efforts have been made to treat patients using DBS. Yet, clinical studies thus far are not very promising. Caston et al. provide a complete overview on the cerebral pain network and potential targets for DBS [5]. The authors put forward a novel avenue to define potential DBS targets. They postulate combining electrocorticography or stereo-EEG (sEEG) with the thermal grill illusion method to map the cerebral pain network to depict possible targets. An interesting approach suggested by Shirvalkar and colleagues is to use sEEG [6]. They propose a trial period in which the electrodes are initially externalized. The effect of stimulation can then first be evaluated before a complete DBS system is implanted. Moreover, this strategy gives the opportunity to obtain neural signals, which might be used as biomarkers of stimulation-induced pain relief. 

The most widely used indications for DBS remain Parkinson’s disease (PD) and essential tremor (ET). DBS is considered a standard treatment for these disorders. Still, there is plenty of room for improvement. In this prospect, Bogdan and colleagues propose a novel individualized approach to optimize DBS settings for patients with ET [7]. Optimal tremor control can be achieved in patients who do not respond to conventional DBS settings or show habituation. Therefore, commercial DBS parties need to provide access for clinicians to stimulation options beyond the standard set of stimulation settings. 

Traditionally, DBS electrodes are implanted whilst using local analgesia on the patient. A trend is to conduct DBS surgeries under general anesthesia [8]. Park and colleagues argue that the classical view, that DBS surgeries for PD patients are best performed in an awake condition to conduct micro-electrode recordings (MER) and macrostimulation, is ready for a change. Their literature review forms the basis to initiate non-inferiority studies to confirm the safety and efficacy of DBS surgeries under general anesthesia. In our electrophysiology study, we studied the effects of procedural sedation and analgesia (PSA) on MER [9]. One of our main findings was that dexmedetomidine reduces the power of the multi-unit activity (MUA) in a dose-dependent matter. The power of the MUA is a parameter which is commonly used to identify the subthalamic nucleus (STN). To what extent the use of anesthetics alters the MER signal that hampers accurate identification of the STN needs further study. Of utmost importance to achieve an optimal lead placement is the quality of the planning based on magnetic resonance imaging (MRI). Our imaging group provided a critical viewpoint on the optimization of pre-operative imaging at the level of acquisition, data-processing and planning software [10]. The individual success of DBS relates considerably to psychological aspects. Baertschi et al. show that illness representations and coping strategies in PD patients are not changed by DBS. However, psychological variances between PD patients should be considered in the acceptation process of life with DBS [11]. Finally, we are challenged by Artusi and colleagues to advance our decision making in the selection process for DBS in PD patients. The current clinical assessment could well benefit from decision support systems, including well-defined phenotypic as well as genetic aspects [12]. 

The novel concepts to optimize DBS have been developed in a fascinating rally over recent decades. Imaging techniques, using high-field MRI, have evolved considerably. New generations of DBS systems offer more programming options, thereby expanding the therapeutic window. The failure of randomized controlled DBS trials for several different disorders is reflected by the critical reviews presented in this Special Issue. A trend towards a different scientific policy using a more individualized approach for each patient will open new avenues in the field of DBS, while further neurotechnical advances may, in the future, allow DBS or alternatives to DBS to be offered to a broader group of patients. To conclude, we endorse a mechanism-based approach using translational research programs involving diverse experts ranging from basic neuroscientist, engineers, ethicists and clinicians to advance the field of DBS.
